# Efficient Synthesis and Antimicrobial Evaluation of Pyrazolopyranopyrimidines in the Presence of SBA-Pr-SO3H as a Nanoporous Acid Catalyst

**Published:** 2018

**Authors:** Ghodsi Mohammadi Ziarani, Faezeh Aleali, Negar Lashgari, Alireza Badiei, Ali Abolhasani Soorki

**Affiliations:** a *Department of Chemistry, Alzahra University, Tehran, Iran. *; b *School of Chemistry, College of Science, University of Tehran, Tehran, Iran. *; c *ACECR-Research Institute of Applied Sciences, Shahid Beheshti University, Tehran, Iran.*

**Keywords:** SBA-Pr-SO_3_H, Green synthesis, Antimicrobial activity, Barbituric acid, Pyranopyrimidine

## Abstract

A simple, efficient, and environmentally friendly method has been developed for the synthesis of a series of tricyclic fused pyrazolopyranopyrimidines via a one-pot three-component reaction of barbituric acids, aromatic aldehydes, and 3-methyl-5-pyrazolone in the presence of SBA-Pr-SO_3_H. SBA-15 mesoporous silica material functionalized with propyl sulfonic acid groups was used as a heterogeneous Brønsted acid catalyst with hexagonal structure, high surface area, thick walls, and large uniform pores. All reactions were performed under reflux conditions in water in the presence of a catalytic amount of SBA-Pr-SO_3_H. High yields, mild reaction conditions, short reaction times, and simple work-up procedures are some advantages of this method. The antimicrobial activities of the synthesized compounds were also evaluated and some products exhibited significant antibacterial activities at low concentrations.

## Introduction

**Figure 1:**
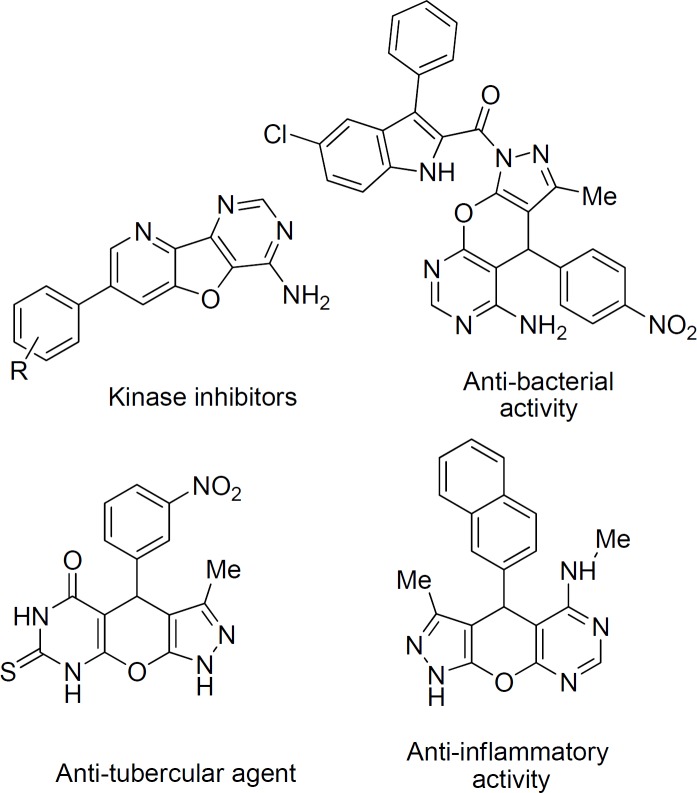
Representative examples of bio-active derivatives of fused pyrimidines

Heterocyclic compounds containing pyran, pyrimidine, and pyrazole moieties have been found to possess various biological and pharmacological properties. The fused pyran ring is a prominent structural motif found in a variety of bioactive compounds such as anticoagulant (1), anticancer (2) antimicrobial (3) antimalarial (4, 5) anti-HIV (6) and anti-fungal agents (7). Substituted pyrimidines constitute a class of biologically important compounds showing analgesic and anti-inflammatory activities (8, 9) and also act as potential kinases inhibitors (10, 11). Furthermore, pyrazole derivatives are important structural subunits with a broad range of pharmacological properties such as anticancer (12) antibacterial (13) analgesic (14) and anti-inflammatory activities (15). Pyranopyrazoles and fused pyrimidines especially pyrazolopyranopyrimidine derivatives exhibit unique potential anti-inflammatory, analgesic, antipyretic, and anti-tubercular activities (Figure 1) (16-18).

In modern organic chemistry, considerable efforts have been devoted to the design of strategies leading to structurally diverse and complex molecules (19-21). In this context, multicomponent reactions (MCRs) offer significant advantages over conventional linear-type syntheses. Such protocols allow molecules to be assembled from three or more starting materials in a one-pot process (22, 23). 

Propyl sulfonic acid functionalized SBA-15 as a heterogeneous Brønsted acid with a hexagonal structure, high surface area, and large pore size exhibits efficient catalytic activity in a variety of organic reactions (24). In continuation of our previous studies on the application of nanoporous solid catalysts in organic reactions (25-28) herein, we report on the design and optimization of a convenient MCR approach for the synthesis of pyrazolopyranopyrimidine derivatives using SBA-Pr-SO_3_H as a nano-catalyst. Some of the synthesized compounds displayed significant antimicrobial activity against some fungi and gram positive and negative bacteria.

## Experimental

Chemical compounds employed in this work were purchased from Merck Company and used with no purification. IR spectra of samples were recorded on FT-IR Bruker Tensor 27 instrument. Melting points were measured using the capillary tube method with an Electrothermal 9200 apparatus. The ^1^H (250 MHz) and ^13^C NMR (62.5 MHz) were run on a Bruker DPX using tetramethylsilane (TMS) as an internal standard (DMSO-d_6_ solution). Mass spectra data were obtained using the network mass selective detector (Agilent) 6890/5973. SEM analysis was undertaken on a Philips XL-30 field-emission scanning electron microscope operated at 16 kV. TEM analysis was performed on a Tecnai G^2^ F30 at 300 kV.

*Preparation of catalyst*

The nanoporous compound SBA-15 was synthesized and functionalized according to a previous report (29) and the modified SBA-Pr-SO_3_H was used as a catalyst in the following reaction.

**Table 1:** The optimization of reaction condition in the synthesis of pyrazolopyranopyrimidine.^a^

Entry	solvent	Time (min)	Yield (%)^b^
1	H_2_O (reflux)	10	92
2	H_2_O (r.t.)	30	63
3	Neat (110°C)	15	80
4^c^	H_2_O (reflux)	60	< 30

a Reaction conditions: barbituric acid (1 mmol), 4-chlorobenzaldehyde (1 mmol), 3-methyl-5-pyrazolone (1mmol), and SBA-Pr-SO_3_H (0.02 g).

b Isolated yield

c Catalyst-free

**Table 2:** Synthesis of pyrazolopyranopyrimidines in the presence of SBA-Pr-SO_3_H under reflux conditions.

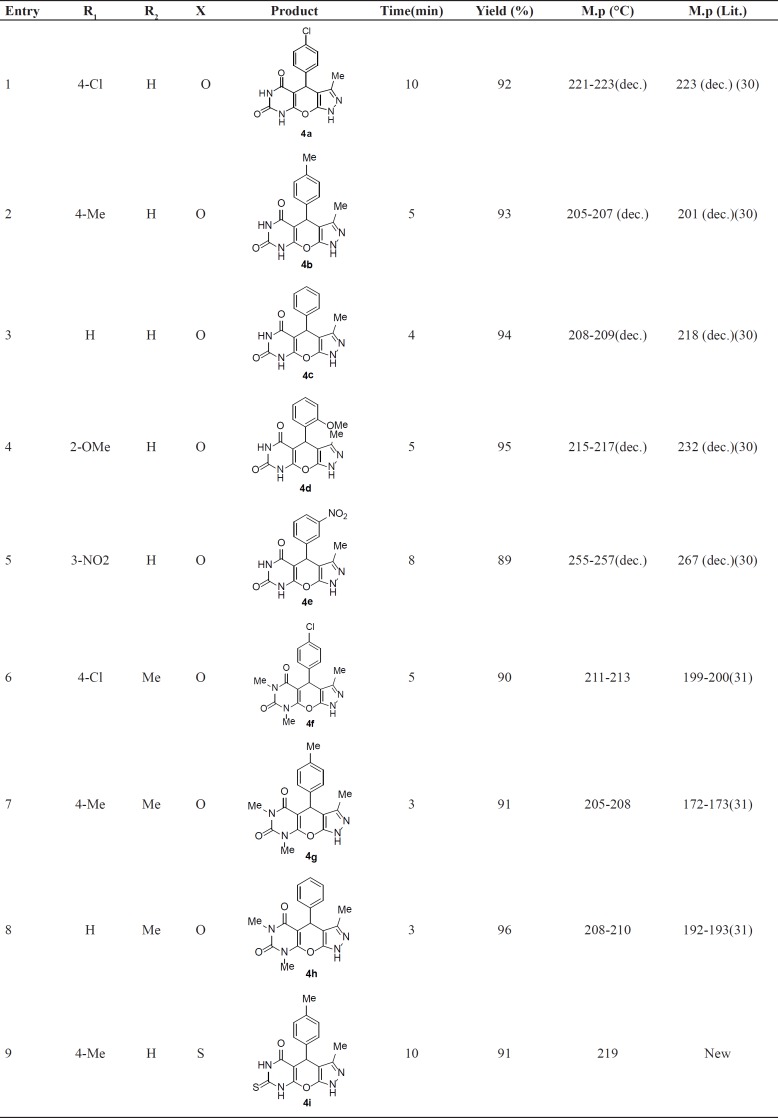

**Table 3:** Comparison of efficiency of various catalysts in the synthesis of pyrazolopyranopyrimidines

**Entry**	**Catalyst**	**Solvent**	**Condition**	**Time (min)**	**Yield (%)**	**Year**
1	DABCO	H_2_O	Reflux	20-45	84-99	2014 (30)
2	Meglumine	H_2_O	Stir. (r.t.)	15-60	89-95	2014 (31)
3	SBA-Pr-SO_3_H	H_2_O	Reflux	3-10	89-96	This work

**Table 4:** Inhibition zone (mm) of synthesized compounds against some gram positive and gram negative bacteria and fungi, by disc diffusion method (IZ = 250 µg/disc).

**Compound**	***B. subtilis***	***S. aureus***	***E. coli***	***P. aeruginosa***	***C. albicans***
**4a**	**20**	**24**	**12**	**0**	**28**
**4b**	**18**	**22**	**12**	**0**	**20**
**4c**	**15**	**21**	**13**	**0**	**20**
**4d**	**14**	**19**	**12**	**0**	**20**
**4e**	**16**	**24**	**0**	**0**	**0**
**4f**	**21**	**24**	**12**	**0**	**26**
**4g**	**19**	**23**	**14**	**0**	**20**
**4h**	**21**	**25**	**16**	**0**	**24**
**4i**	**16**	**20**	**12**	**0**	**20**
**Chloramphenicol**	26	22	24	8	-
**Gentamicin**	28	20	20	18	-
**Nystatin**	-	-	-	-	18

**Table 5:** Minimum inhibitory concentration (µg/mL) of synthesized compounds against some gram positive and gram negative bacteria and fungi

**Compound**	***B. subtilis***	***S. aureus***	***E. coli***	***P. aeruginosa***	***C. albicans***
**4a**	32	8	256	-	2
**4b**	64	16	256	-	32
**4c**	128	32	128	-	32
**4d**	128	32	256	-	32
**4e**	-	-	-	-	-
**4f**	16	8	256	-	4
**4g**	32	16	128	-	32
**4h**	16	8	64	-	8
**4i**	-	-	-	-	-
**Chloramphenicol**	4	8	4	256	-
**Gentamicin**	0.125	0.5	0.5	1	-
**Nystatin**	-	-	-	-	8

**Scheme 1:**
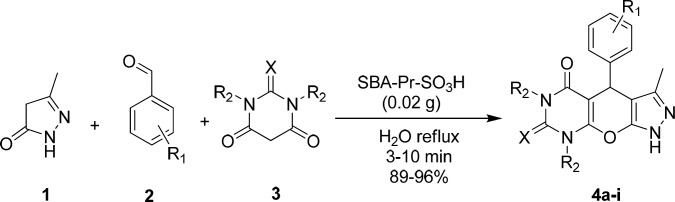
Synthesis of pyrazolopyranopyrimidine derivatives **4a-i** in the presence of SBA-Pr-SO_3_H.

**Scheme 2:**
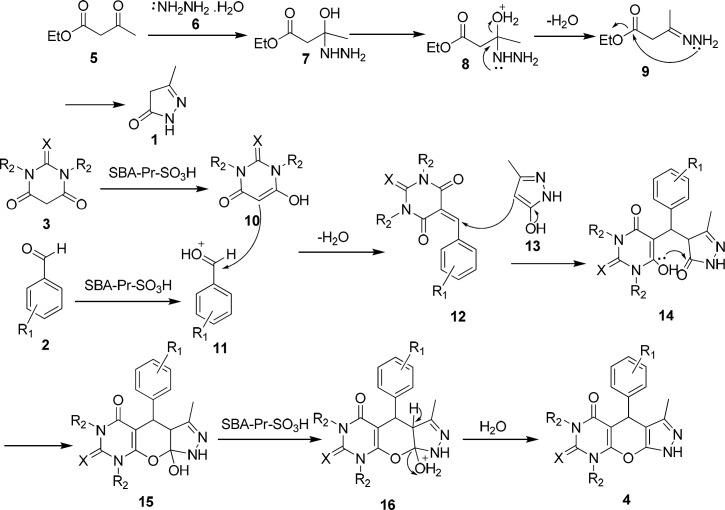
Proposed mechanism

**Figure 2:**
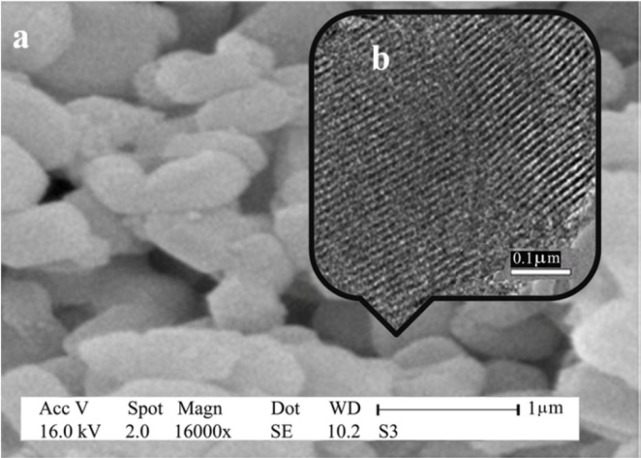
SEM (a) and TEM (b) images of SBA-Pr-SO_3_H

**Figure 3:**
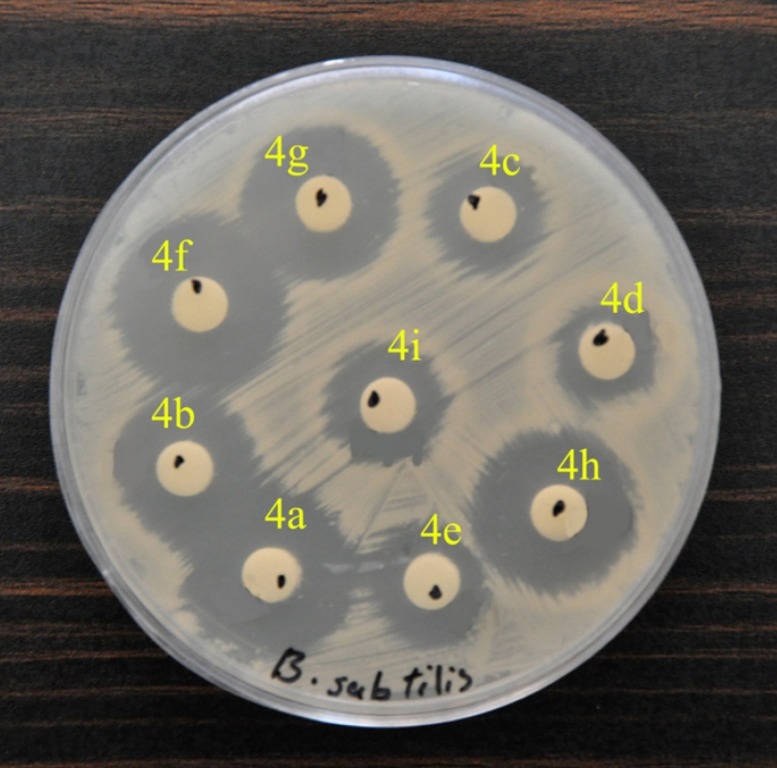
Disk-diffusion testing of antimicrobial susceptibility to the synthesized compounds

*Procedure for the synthesis of 3-methyl-5-pyrazolone*

A solution containing hydrazine hydrate 80% (1.4 mmol, 0.07 g) and ethyl acetoacetate (1 mmol, 0.13 g) in ethanol (10 mL) was stirred at room temperature for 5 min. After the completion of reaction as indicated by TLC, the solution was diluted with ethanol (10 mL) and stirred in an ice bath for 30 min. The resultant solid was then filtrated, washed with cold ethanol, and recrystallized from ethanol to give pure 3-methyl-5-pyrazolone. 

*General procedure for the synthesis of pyrazolopyrznopyrimidines*

A mixture of barbituric acid (1 mmol, 0.128 g), aromatic aldehydes (1 mmol), 3-methyl-5-pyrazolone (1 mmol, 0.098 g), and SBA-Pr-SO_3_H (0.02 g) was refluxed in water (4 mL) for the appropriated length of time. After completion of the reaction (monitored by TLC), the generated solid product was dissolved in hot ethanol and acetone (1:1), the heterogeneous solid catalyst was insoluble and could be removed by filtration. The pure products 4a-i were obtained after cooling of the filtrates. The catalyst was washed with diluted acid solution, water, and then acetone, dried under vacuum and reused for several times. The physical and spectral data of new compounds are given below: 

*3,6,8-Trimethyl-4-phenyl-6,8-dihydropyrazolo[4* ^ꞌ^ *,3* ^ꞌ^ *:5,6]pyrano-[2,3-d]pyrimidine-5,7(1H,4H)-dione (4h)*

White solid, Yield: 96%, M.p. 208-210. IR (KBr) *ν*: 2923, 1683, 1572, 1468, 1386, 1323, 1276, 1235, 1142, 822, 698 cm^-1^. ^1^H NMR (250 MHz, DMSO-d_6_) *δ*: 2.23 (s, 3H, CH_3_), 3.11 (s, 6H, CH_3_), 5.54 (s, 1H, CH), 7.00-7.19 (m, 5H, ArH), 13.5 (br s, 1H, NH) ppm. ^13^C NMR (62.5 MHz, DMSO-d_6_) *δ*: 10.4, 28.2, 32.5, 91.6, 106.3, 125.7, 127.1, 128.3, 142.8, 144.2, 152.1, 159.5, 163.7 ppm. Mass *m/z* (%): 324 (14), 243 (100), 186 (73), 156 (58), 42 (91). Anal. Calcd for C_17_H_16_N_4_O_3_: C, 62.95; H, 4.97; N, 17.27. Found: C, 62.88; H, 5.05; N, 17.21.

*3-Methyl-7-thioxo-4-(p-tolyl)-4,6,7,8-tetrehydropyrazolo[4ꞌ,3ꞌ:5,6]pyrano[2,3-d]pyrimidin-5(1H)-one (4i)*

White solid, Yield: 91%, M.p. 219 °C. IR (KBr) *ν*: 3421, 2922, 1623, 1533, 1508, 1224, 649, 511 cm^-1^. ^1^H NMR (250 MHz, DMSO-d_6_) *δ*: 2.18 (s, 3H, CH_3_), 2.21 (s, 3H, CH_3_), 5.37 (s, 1H, CH), 6.88 (d, *J* = 7.7 Hz, 2H, ArH), 6.98 (d, *J* = 7.7 Hz, 2H, ArH), 11.49 (s, 2H, NH), 13.48 (br s, 1H, NH) ppm. ^13^C NMR (62.5 MHz, DMSO-d_6_) *δ*: 10.4, 20.9, 30.6, 96.6, 106.0, 126.9, 128.9, 134.8, 139.0, 139.1, 144.2, 159.6, 159.7, 163.9, 173.4 ppm. Mass *m/z* (%): 326 (2.5), 281 (2.5), 246 (20), 199 (100), 185 (71), 115 (59). Anal. Calcd for C_16_H_14_N_4_O_2_S: C, 58.88; H, 4.32: N, 17.17. Found: C, 58.79; H, 4.23: N, 17.25.

*General procedure for in-vitro antibacterial evaluation of compounds 4a-i*

The biological activities of compounds 4a-i were screened *in-vitro* using the disc diffusion method (IZ) and subsequently the minimum inhibitory concentration method (MIC). The microorganisms used were *Pseudomonas aeruginosa* (ATCC 85327) and *Escherichia coli* (ATCC 25922) as gram-negative bacteria,* Staphylococcus aureus* (ATCC 25923) and *Bacillus subtilis* (ATCC 465) as gram-positive bacteria, and *Candida albicans* (ATCC 10231) as the fungus. All obtained compounds were dissolved in DMSO (100 µg/mL) and 25 µL was loaded onto 6-mm paper discs. One hundred microliters of 10^9^ cell/mL suspension of the microorganisms was spread on sterile Mueller–Hinton agar plates, and the discs were placed on the surface of culture plates. The MIC of the synthesized compounds which showed antibiotic activity in disc diffusion tests was also determined by microdilution method and activities of each compound were compared with chloramphenicol, gentamicin, and nystatin as references.

## Results and Discussion

Herein, we report a green and highly efficient method for the synthesis of pyrazolopyranopyrimidine derivatives through the one-pot condensation of 3-methyl-5-pyrazolone 1 (1 mmol), aromatic aldehydes 2 (1 mmol), barbituric acid 3 (1 mmol), and SBA-Pr-SO_3_H as the heterogeneous catalyst in water (Scheme 1). 3-Methyl-5-pyrazolone 1 was synthesized in the way that was mentioned in experimental section. To optimize the reaction conditions, barbituric acid, 4-chlorobenzaldehyde, and 3-methyl-5-pyrazolone in a molar ratio of (1:1:1) were selected as a model reaction. Comparing the reaction times and yields of products under different conditions such as using water as the solvent (under reflux and room temperature) and also solvent free condition, the best result (92% yield) was obtained under reflux conditions in water after 10 min in the presence of SBA-Pr-SO_3_H (0.02 g). As shown in Table 1, the presence of the catalyst was found to give a higher yield of products in reasonable reaction times. In contrast, in the absence of any catalyst in water, this reaction afforded compound 4a after 60 min in very low yield (< 30%).

To evaluate the scope and generality of this protocol, different aromatic aldehydes and barbituric acids were used in the presence of SBA-Pr-SO_3_H as the catalyst. The results are summarized in Table 2. Corresponding pyrazolopyranopyrimidines were successfully prepared in high to excellent yields. As shown in Table 2, both electron-rich and electron-deficient aldehydes gave pyrazolopyranopyrimidines in excellent yields and very short reaction times. Melting points were compared with reported literature values (Table 2). The generated solid products were dissolved in hot ethanol and acetone (1:1), and separation of the heterogeneous solid catalyst from the reaction medium was easily carried out by simple filtration.

The reactions were monitored by TLC. The generated solid product was dissolved in hot ethanol and acetone (1:1), filtered for removing the catalyst and then the filtrate was cooled to afford the pure crystals of pyrazolopyranopyrimidines. 

A possible mechanism for the formation of products 4a-i in the presence of SBA-Pr-SO_3_H is shown in Scheme 2. At first, 3-methyl-5-pyrazolone 1 was obtained through the nucleophilic attack of hydrazine hydrate 6 to ethyl acetoacetate 5 to form an imine intermediate 9 which further undergoes intermolecular condensation to form 3-methyl-5-pyrazolone 1. In the main reaction, protonation of the carbonyl group of benzaldehyde 2 by the solid acid catalyst activates it toward nucleophilic attack of barbituric acid enole form 10 to yield intermediate 12. Subsequently, the Michael addition of 3-methyl-5-pyrazolone enolic form 13 to compound 12 results in the formation of intermediate 14 which by the cycloaddition of hydroxyl group to the carbonyl group produces compound 15. Finally, elimination of water affords the corresponding pyrazolopyranopyrimidine 4.

Table 3 compares the effectiveness of various catalysts used in the synthesis of pyrazolopyranopyrimines. The results demonstrate that the present methodology is more efficient and less time-consuming when compared with other methods.

The morphology of SBA-Pr-SO_3_H was verified by SEM and TEM images (Figure 2). The results confirmed that the hexagonally ordered mesoporous structure of SBA-15 silica was well retained after the chemical grafting reaction. The TEM image (Figure 2b) showed the uniform and parallel channels, which were open along the particles.

The inhibition zones of compounds around the discs are shown in Table 4. As can be seen, all compounds exhibited significant antibacterial activities against *S. aureus* and *C. albicans *when compared with the reference drugs. All compounds were able to inhibit the growth of *B. subtilis* and *E. coli*. Figure. 3 illustrates the inhibition zones of compounds around the disks with *B. subtilis*. No compound showed antibiotic activity against *P. aeruginosa*. Table 5 illustrates the minimum inhibitory concentration (MIC) of the synthesized compounds. The screening results indicated that among the synthesized products, compounds 4a (R_1_ = 4-Cl, R_2_ = H), 4f (R_1_ = 4-Cl, R_2_ = Me)_, _and 4h (R_1_ = H, R_2_ = Me) exhibited significant antibacterial activities at low concentrations, whereas the other compounds generally showed lower activities. The best result was observed for *C. albicans *which was sensitive to compounds 4a, 4f and 4h with MIC being between 2 and 8 µg/mL.

## Conclusion

In conclusion, we have developed an efficient, clean and simple procedure for the synthesis of pyrazolopyranopyrimidine derivatives in excellent yields in the presence of catalytic amount of SBA-Pr-SO_3_H as an environmentally benign solid acid catalyst. High yields, short reaction times, convenient work-up procedure, and the use of water as a solvent are the advantages of this method. Compounds synthesized by this method were screened for their antimicrobial activities and products 4a, 4f and 4h showed significant antimicrobial activities against some fungi and gram positive and gram negative bacteria.
